# Infection Risk Prediction Model for COVID-19 Based on an Analysis of the Settlement of Particles Generated during Dental Procedures in Dental Clinics

**DOI:** 10.1155/2021/7832672

**Published:** 2021-12-30

**Authors:** Paula Alejandra Baldion, Henry Oliveros Rodríguez, Camilo Alejandro Guerrero, Alberto Carlos Cruz, Diego Enrique Betancourt

**Affiliations:** ^1^Departamento de Salud Oral, Facultad de Odontología, Universidad Nacional de Colombia, Bogotá, Colombia; ^2^Departamento de Epidemiología, Facultad de Medicina, Universidad de La Sabana, Chía, Cundinamarca, Colombia

## Abstract

**Background:**

The health emergency declaration owing to severe acute respiratory syndrome coronavirus-2 (SARS-CoV-2) has drawn attention toward nosocomial transmission. The transmission of the disease varies depending on the environmental conditions. Saliva is a recognized SARS-CoV-2 reservoir in infected individuals. Therefore, exposure to fluids during dental procedures leads to a high risk of contagion.

**Objective:**

This study aimed to develop an infection risk prediction model for COVID-19 based on an analysis of the settlement of the aerosolized particles generated during dental procedures.

**Materials and Methods:**

The settlement of aerosolized particles during dental aerosol-generating procedures (AGPs) performed on phantoms was evaluated using colored saliva. The gravity-deposited particles were registered using a filter paper within the perimeter of the phantom head, and the settled particles were recorded in standardized photographs. Digital images were processed to analyze the stained area. A logistic regression model was built with the variables ventilation, distance from the mouth, instrument used, area of the mouth treated, and location within the perimeter area.

**Results:**

The largest percentage of the areas stained by settled particles ranged from 1 to 5 *µ*m. The maximum settlement range from the mouth of the phantom head was 320 cm, with a high-risk cutoff distance of 78 cm. Ventilation, distance, instrument used, area of the mouth being treated, and location within the perimeter showed association with the amount of settled particles. These variables were used for constructing a scale to determine the risk of exposure to settled particles in dentistry within an infection risk prediction model.

**Conclusion:**

The greatest risk of particle settlement occurs at a distance up to 78 cm from the phantom mouth, with inadequate ventilation, and when working with a high-speed handpiece. The majority of the settled particles generated during the AGPs presented stained areas ranging from 1 to 5 *µ*m. This model was useful for predicting the risk of exposure to COVID-19 in dental practice.

## 1. Introduction

COVID-19 is an infectious disease caused by coronavirus (severe acute respiratory syndrome coronavirus 2 [SARS-CoV-2]) and has spread worldwide in recent months [1]. As of now, the great majority of countries have already undergone a second wave of coronavirus transmissions. The nosocomial transmission of this infection has been reported more recently [2–4].

The mode of transmission of the disease and the extent of nosocomial infection vary depending on the conditions of the environment [5]. To et al. [6] recognized saliva as a reservoir of SARS-CoV-2 in infected individuals; therefore, it is recommended to reduce the number of aerosol-generating procedures (AGPs) during the COVID-19 pandemic. Exposure to saliva produced during the majority of AGPs represents a high risk of contagion in dental schools, clinics, and offices [7–9]. Taking into account that the mouth is part of the oronasal pharynx, it also houses viruses and bacteria from the respiratory tract. Therefore, AGPs may produce aerosolized saliva and then lead to airborne spreading of infections such as SARS-CoV-2 [10].

According to the guidelines set by the World Health Organization and various other government agencies for the gradual restoration of health services during the mitigation and control phases of the current health emergency, infection in oral health care settings can be transmitted directly through the inhalation of droplets generated while coughing or sneezing, by the exposure of the mucous membrane to infectious droplets, and by indirect transmission through contaminated surfaces [11, 12]. Transmission of SARS-CoV-2 may occur when droplets (diameter, >5 *µ*m) that do not remain suspended in the air are deposited on surfaces at a distance of <2 m from the individual; contact with these surfaces can contaminate the mucosa of the mouth, nose, or conjunctiva [13, 14].

Although different transmission mechanisms have been proposed, the airborne transmission is the dominant route of transmission [15]. Airborne transmission may occur when tiny respiratory droplets are desiccated to 20–40% diameter; this residual is called droplet nuclei with a wide diameter range (<100 *µ*m) [16], and which could contain viral particles. Droplet nuclei are residues of an evaporated droplet [17] and can remain suspended in the air for long periods like cloud condensation nuclei [18], reaching people more than one meter away [19]. Airborne transmission of the SARS-CoV-2 virus may be possible under specific circumstances and in the locations where AGPs are performed (during manual ventilation before intubation, intubation, tracheostomy, and open airway suction) [20, 21]. The risk in dentistry is mainly related to the use of high-speed handpieces and ultrasound instruments, which generate aerosol particles often mixed with saliva and blood [22, 23].

Even though the effectiveness of vaccination has been now established, the duration of the vaccine-induced immunity remains unknown [24]. Thus, infection control measures such as personal protective equipment (PPE) remain necessary in order to protect patients and health care providers. In turn, those measures should be adjusted to meet the needs of each setting. Presuming that COVID-19 will remain a pandemic disease for a significant amount of time and that, therefore, there are still several waves to come, it is necessary to effectively increase biosafety at oral health care settings in order to face the infection risk properly. Hence, the objective of this study was to develop a model for predicting the risk of exposure to SARS-CoV-2 through the description of the settlement of AGP-generated particles onto surfaces and the definition of the corresponding settlement patterns.

## 2. Methods

### 2.1. Spread of Particles by Aerosol-Generating Instruments

#### 2.1.1. Location and Distribution of Experimental Units

**Figure 1:**
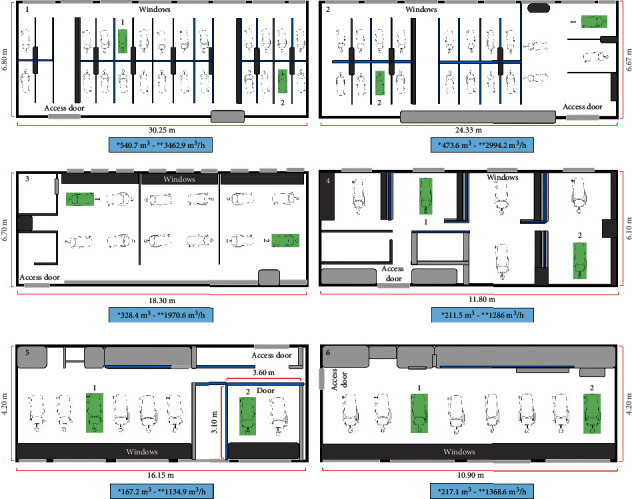
Location of the experimental units in dental clinics. (1, 2, 3, and 5) General and prosthodontics dentistry clinic. (4) Periodontics clinic. (6) Orthodontics clinic. The location of the dental units where the experiments were performed is highlighted in green. Unit 1, adequate ventilation. Unit 2, inadequate ventilation. Values in blue boxes: ^*∗*^Room volume (m^3^), ^*∗∗*^Air-exchange rate in room with open windows (m^3^/h).

After receiving approval from the Institutional Ethics Committee (B-CIEFO-074-2020), two dental units were selected from each one of the six dental clinics from the Faculty of Dentistry of the National University of Colombia. Phantom heads (Bader, Nigran, Pontevedra, Spain) were assembled in each dental unit, and an ANA-4 typodont (Bader) containing 28 teeth was installed in each phantom. Phantom's heads were always placed at a 90 cm height. The phantoms were placed in two distant areas in the clinics to evaluate the influence of changes in airflow generated within the clinics, unit with adequate ventilation (Unit 1) and unit with inadequate ventilation (Unit 2) (Figure 1). Regarding ventilation, the most convenient location of the dental unit with respect to the windows and to the access of each clinic (Unit 1) was determined using a portable smoke machine (AGPTEK 500W, NY, USA) with a power of 2000 CFM output. A range of 3 m and a fog time duration of 25 s were used, to evaluate the generation of an unidirectional airflow without cloud formation or turbulence to find the dental unit where it would have the best ventilated area in each clinic. To verify the ventilation conditions with open windows, the air-exchange rate in the room was measured in each clinic using an anemometer HT625A HABOTEST (Dongguan, China) (Figure 1), and the room volume was also calculated. None of the clinics has any vents or air extraction system, only natural ventilation.

#### 2.1.2. Aerosol-Generating Dental Procedures

**Figure 2:**
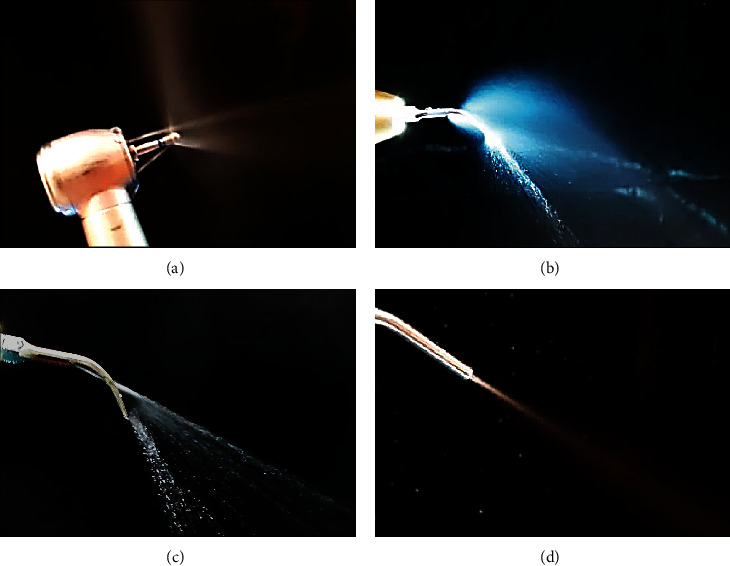
Aerosol-generating instruments used in the study. (a) Dental high-speed handpiece (KaVo, Standard head, Triple water spray, Berlin, Germany), with an irrigant flow rate of 50 mL/min. (b) Pneumatic scaler (AS2000, NSK, Shimohinata, Kanuma, Japan), with an irrigant flow rate of 55 mL/min. (c) Ultrasonic scaler (Cavitron Bobcat Pro, Dentsply, NY, USA), with an irrigant flow rate of 55 mL/min. (d) Air-water syringe, with an irrigant flow rate of 50 mL/min.

**Figure 3:**
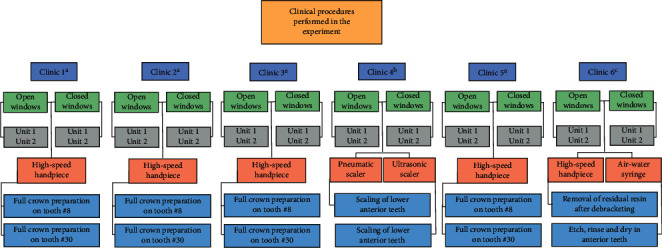
Summary of the clinical procedures performed in the study. ^a^General and prosthodontics dentistry clinic. ^b^Periodontics clinic. ^c^Orthodontics clinic. Each procedure was performed for 12 min.

Dental procedures were performed using four aerosol-generating instruments: high-speed handpiece, pneumatic scaler, ultrasonic scaler, and triple syringe. The irrigant flow rate of all instruments was calibrated to obtain values between 50 and 55 mL/min, and it was measured with a graduated glass (Figure 2). These instruments were used in a total of 48 procedures and sorted as follows: eight procedures per clinic in six clinics, corresponding to two procedures replicated in two different placements within each clinic, and in two different conditions of ventilation—open window and closed window—(Figure 3).

In the four general dentistry and prosthodontic clinics, a high-speed handpiece was used to make full crown preparations on a given anterior tooth (tooth #8) and full crown preparations on a given posterior tooth (tooth #30). An expert operator performed the procedures in a standardized way with regard to the dimensions and the time required for the treatment. The preparation for a full ceramic crown (1.8 mm deep with a 6° convergence) was performed with a medium-grit, flat-end tapered diamond bur (Brasseler, Savannah, GA, USA). Palatal reduction on the anterior tooth was performed using the egg-shaped diamond instrument (Brasseler). The final preparation was refined with matching finishing diamonds (Brasseler). Adequate and even tooth reduction was checked with a silicone matrix. In the periodontics clinic, two scaling of lower anterior teeth procedures were performed to compare the stained area produced by settled particles generated by the pneumatic scaler and the ultrasonic scaler. Finally, in the orthodontics clinic, two procedures commonly carried out for assembling and disassembling orthodontic appliances were performed, namely, removal of residual resin after debracketing using matching finishing diamonds (Brasseler) followed by etching, rinsing, and drying performed on anterior using an air-water syringe. The operator and the assistant used the recommended PPE comprising a long-sleeved surgical gown with back closure, disposable cap, latex gloves, N-95 mask, eye protector, face shield, and closed shoes.

#### 2.1.3. Collection of Samples

**Figure 4:**
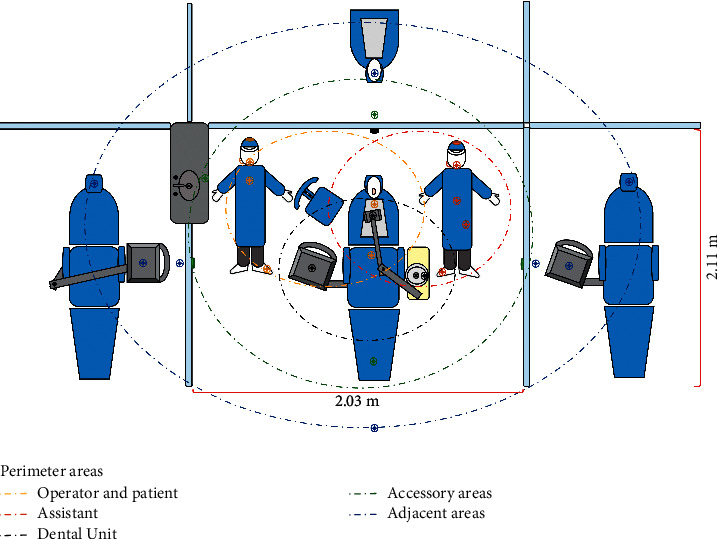
Filter paper placement per dental unit and delimitation of perimeter areas for particles dispersion analysis. Colored dash-dotted ovals delimit the five perimeter areas observed: perimeter area of the operator and patient, perimeter area of the assistant, perimeter area of the dental unit, accessory areas, and adjacent areas. Circled crosses indicate the placement of each plastic Petri dish within each perimeter area. For each procedure, 26 filter papers were located within the perimeter area of each dental unit in each clinic, except for Clinic 1, where 27 filter papers were placed. These add up to a total of 1256 samples.

A nontoxic concentrated artificial dye (Nature's Flavors, Orange, CA, USA) diluted 1 : 100 in water was loaded into the accessory tank of two dental units in each clinic. In addition, artificial saliva was prepared using propylene-glycol (2.2 mL), carboxymethylcellulose (14 g), NaCl (0.58 g), CaCl_2_ (0.17 g), and MgCl_2_ (0.08 g) in 970 mL of distilled water, which were mixed with 30 mL of artificial dye until a density of 1.005 g/mL was reached. The density was measured using a density meter (DMA 4500, Anton Paar, Hidalgo, Mexico) calibrated at 15.56°C. The colored saliva was poured into the posterior oral area of each phantom head to simulate natural saliva. The gravity-deposited aerosol particles were registered using a filter paper placed in plastic Petri dishes (size, 100 × 25 mm). Different areas within the perimeter of the phantom head (up to 320 cm away from each phantom's mouth) were covered to obtain 1256 samples collected from settled aerosol particles in the six clinics (Figure 4). The papers were held in position for up to 30 min after the end of the procedure. The average ambient temperature in the clinical areas was 20°C, and the relative humidity was 70%. A 2 : 1 air-water pressure ratio was used in each dental unit. The supply pressures for air and water were calibrated at 80 psi and 40 psi, respectively.

#### 2.1.4. Identification of Gravity-Deposited Particles

**Figure 5:**
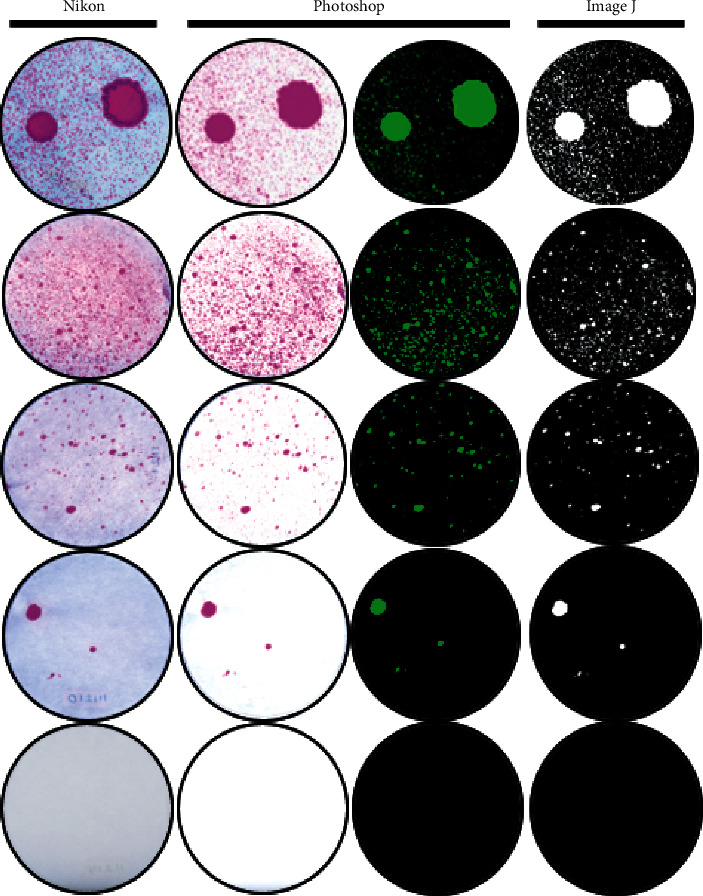
Image processing to analyze the stained area of the settled particles. The stained area was defined as the sum of the stained area measurement of each settled particle on the filter paper in *µ*m^2^.

The dispersion of the dye on the filter paper was recorded using standardized photographs of the stained areas. The photos were taken with a Nikon D5500 camera (Nikon Corporation, Tokyo, Japan) and standardized at a focal length of 85 mm | 6000 × 4000 dimensions | 1/125 s |f/32 | ISO 200. To improve the detection of the settled particles on the filter paper, colorimetric images were converted into fluorometric-type images using the invert and color balance tools on Adobe Photoshop software (Adobe Inc, San Jose, CA, USA). The digital images were processed to analyze the stained area using the ImageJ program (National Institutes of Health, Bethesda, MD, USA) in a binary system (Figure 5) in order to determine the number and size of the particles. The sizes of the settled aerosol particles were measured in pixels and converted to microns (*µ*m).

### 2.2. Data Analysis

The analyses were carried out using the statistical package SAS 9.3 (SAS Institute, Cary, North Carolina, USA). The Microsoft Office Excel 2010 software (Microsoft Corporation, Redmond, Washington, USA) was used for the construction of the databases. The independent variables—i.e., ventilation, distance from the mouth, instrument used, region in the mouth to be treated, location within the perimeter area, division between units, and placement of the dental unit with regard to the windows—were analyzed using a bivariate analysis in order to determine their association with the “stained area” outcome (*p* < 0.05). The infection risk prediction model for exposure to settled particles included the variables that showed either statistical association or biological plausibility. Normality of the data on the stained area was determined using the Shapiro–Wilk test. The outcome variable “stain area” was dichotomized from the median (50^th^ percentile) after performing a log transformation, whereas the “distance” variable was dichotomized according to the cutoff points established by the maximum discriminatory capacity with respect to the stained area. The odds ratio (OR) values were obtained from the logistic regression model and used to determine the scores of the variables used for the construction of an infection risk scale for COVID-19.

## 3. Results

### 3.1. Spread of Aerosol Particles

**Figure 6:**
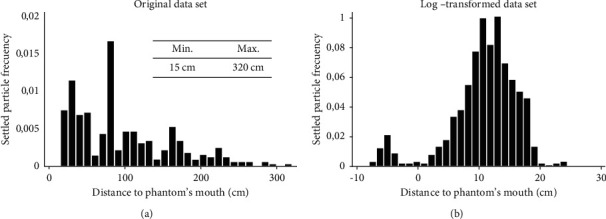
Histograms of original data (a) and log-transformed data (b) of the distribution of settled particles according to the distance.

One thousand two hundred and fifty-six sites were evaluated within the perimeters of the phantom heads, of which 739 had a greater or lesser degree of particles settlement. The most significant particle settlements were located within a perimeter area inferior to 78 cm away from the phantom's mouth, whence 78 cm was determined as the cutoff point with regard to the risk of infection. It is important to underline that the aforementioned perimeter area encompasses the ordinary location of the operator, patient, assistant, and dental unit. Settlement levels of particles were highest in front of the patient and reduced with increasing distance from the mouth. Some settled particles reached the maximum distance measured, i.e., 320 cm, although few reached accessory areas, such as adjacent units, sinks, auxiliary tables, and walls. An inverse relationship was observed between the distance and the settlement of the particles (Figure 6).

**Figure 7:**
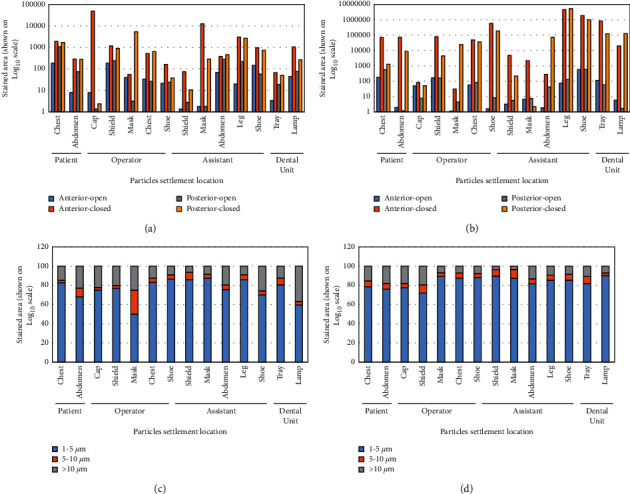
Quantitative analysis of the high-speed handpiece-generated particles settled on surfaces in several locations. “Anterior”: procedures performed in the anterior region of the mouth (full crown preparation on tooth #8). “Posterior”: procedures performed in the posterior region of the mouth (full crown preparation on tooth #30). “Open”: procedures performed with open windows. “Closed”: procedures performed with closed windows. (a) Area stained by the settlement of particles generated in the dental unit with inadequate ventilation (Clinic 3, Unit 2 in Figure 1). (b) Area stained by the settlement of particles generated in the dental unit with adequate ventilation (Clinic 3, Unit 1 in Figure 1). (c) Percentage distribution of stained area generated in the dental unit with inadequate ventilation (Clinic 3, Unit 2 in Figure 1). (d) Percentage distribution of stained area generated in the dental unit with adequate ventilation (Clinic 3, Unit 1 in Figure 1).

The influence of ventilation on the number of settled particles was observed, independent of the location of the dental unit (adequate or inadequate ventilation) from the windows (Figure 7(a) and 7(b)). However, an important decrease in the stained area of the settled particles generated during AGPs performed with the windows opened was observed for the dental unit with adequate ventilation with respect to the unit with inadequate ventilation (Figure 7(a) and 7(b)). Most of the settled particles generated by the high-speed handpiece marked a stained area between 1 and 5 *µ*m (Figure 7(c) and 7(d)).

**Figure 8:**
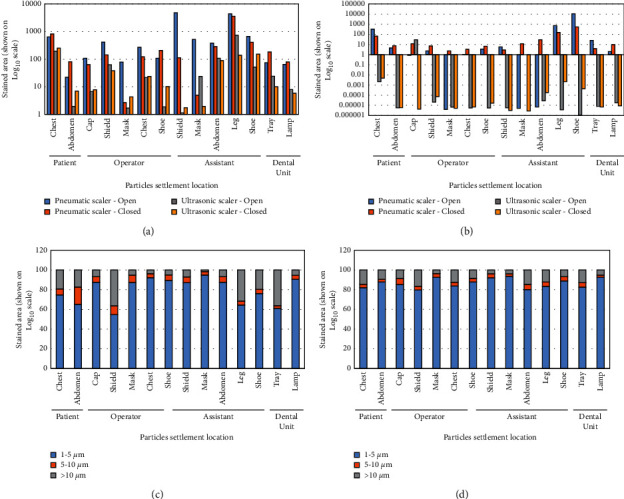
Quantitative analysis of the settled particles generated during scaling of lower anterior teeth in the different sites. “Open”: procedures performed with open windows, and “closed”: procedures performed with closed windows. (a) Stained area of settled particles generated in the dental unit with inadequate ventilation (Clinic 4, Unit 2 in Figure 1) with pneumatic scaler compared to ultrasonic scaler. (b) Stained area of settled particles generated in the dental unit with adequate ventilation (Clinic 4, Unit 1 in Figure 1) with pneumatic scaler compared to the ultrasonic scaler. (c) Percentage distribution of settled particles generated in the dental unit with inadequate ventilation (Clinic 4, Unit 2 in Figure 1). (d) Percentage distribution of settled particles generated in the dental unit with adequate ventilation (Clinic 4, Unit 1 in Figure 1).

The stained areas corresponding to settled particles generated by the sonic and ultrasonic devices were compared. An important influence of ventilation on the stained area was evidenced, especially in the case of particles generated by an ultrasonic scaler (Figure 8(a) and 8(b)). The predominant size of the stained areas of the settled particles remained stable (1 to 5 µm) independent of the instrument (Figure 8(c) and 8(d)). The Shapiro-Wilk normality test demonstrated a nonnormal distribution of the data (*P* < 0.0001). A comparison with the Wilcoxon signed-rank test of the two procedures showed differences between both instruments; the stained area generated by the pneumatic scaler was greater than that produced by the ultrasonic scaler (Table 1).

**Figure 9:**
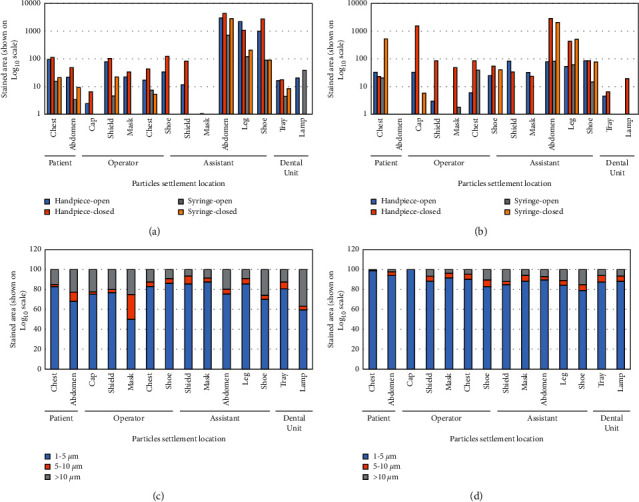
Quantitative analysis of the stained area of settled particles generated during vestibular resin removal and etching, rinsing, and drying in anterior teeth. “Open”: procedures performed with open windows, and “closed”: procedures performed with closed windows. (a) Stained area of settled particles generated in the dental unit with inadequate ventilation (Clinic 6, Unit 2 in Figure 1) with a high-speed handpiece compared to the air/water syringe. (b) Stained area of settled particles generated in the dental unit with adequate ventilation (Clinic 6, Unit 1 in Figure 1) with a high-speed handpiece compared to the air/water syringe. (c) Percentage distribution of settled particles generated in the dental unit with inadequate ventilation (Clinic 6, Unit 2 in Figure 1). (d) Percentage distribution of settled particles generated for the air/water syringe in the dental unit with adequate ventilation (Clinic 6, Unit 1 in Figure 1).

The areas stained by settled particles on the filter papers located near the assistant were greater than those near the operator, patient, and dental unit when working in the anterior teeth (upper and lower) with a high-speed piece and a triple syringe (Figure 9(a)). Airflow through open window ventilation decreased the stained area for both instruments (Figure 9(b)). The stained area produced by the triple syringe (air/water spray) was smaller than those produced by the high-speed handpiece. The mostly settled particles left a stained area between 1 and 5 µm in both instances (Figure 9(c) and 9(d)).

### 3.2. Characterization of Stained Area of Settled Particles Produced during AGPs

The aforementioned AGPs generated a percentage of stained area of particles between 1 and 5 *µ*m higher than those of >5 µm, regardless of the following variables: instrument used, ventilation, distance from the oral cavity, location of the dental unit, and area of the mouth being treated (Table 2).

**Figure 10:**
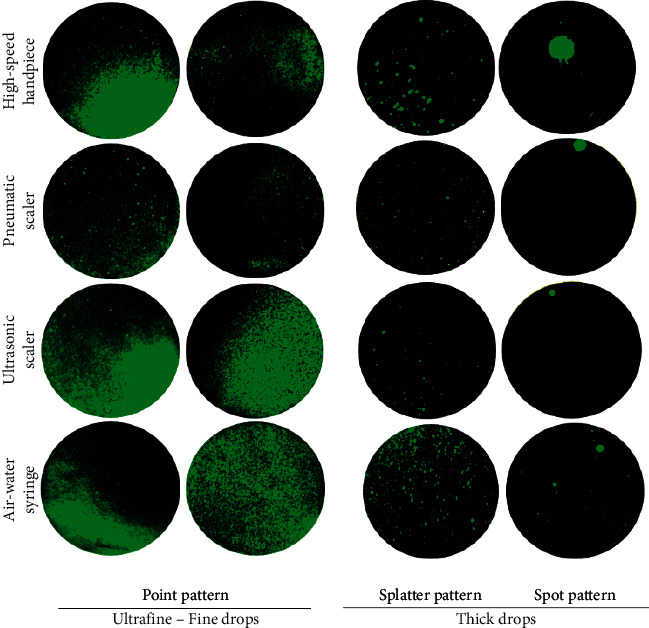
Settlement patterns of particles on filter papers. The four aerosol-generating instruments produced particles that, upon settling, left an area stained with different patterns. Factors such as distance, irrigant flow rate, and filter paper location determine settlement patterns.

The stained area of the settled particles was classified according to the settlement pattern on the surface. Several shape and size patterns were found, depending on the location of the paper from the phantom's mouth, the instrument used, the angle of entry of the drops toward the surface of the paper, and the possibility of the formation of large drops resulting either from the union of several drops before their settlement or from dripping through the gloves or hoses of the dental unit (Figure 10).

### 3.3. Risk Prediction Model

For the construction of the prediction model, the assumptions of normality, independence, and homoscedasticity were verified. Seven variables were used (Table 3), out of which five were selected (ventilation, distance, instrument used, area of the mouth being treated, and location within the perimeter) to be included in the model, based on their association with the stained area outcome and the biological plausibility. For the distance variable, according to its median value, the cutoff point was found at 78 cm away from the mouth. Similarly, the median was obtained as the cutoff point to dichotomize the stained area outcome variable (Table 4).

Five variables were finally included in the model using the forward stepwise method. Considering the more parsimonious model and with a higher coefficient of determination (*R* ^ 2 = 0.3368), the ORs were estimated for each of the variables, as shown in Table 5.

Based on the OR values for the risk of infection due to exposure to aerosol particles generated during AGPs, different scores were assigned to construct the scale, in order to obtain a total value of 10 points, as shown in Table 6.

**Figure 11:**
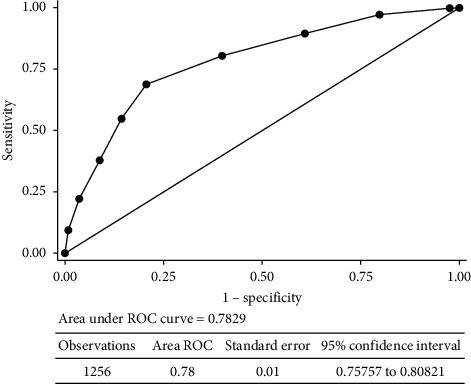
ROC curve of the logistic regression model.

The discrimination capacity of the model was evaluated using an ROC curve (Figure 11). In addition, the fit of the model was evaluated using the Hosmer–Lemeshow test, which demonstrated good discrimination capacity and adequate adjustment, as observed in Table 7. The observed and expected values were reported according to the predictive values obtained by the model for each of the scoring levels. The best cutoff point was five points, which showed adequate sensitivity and specificity (Table 8).

The following scale shows the score assigned to each variable included in the model according to its OR value (Table 9).

### 3.4. Recommendations

**Figure 12:**
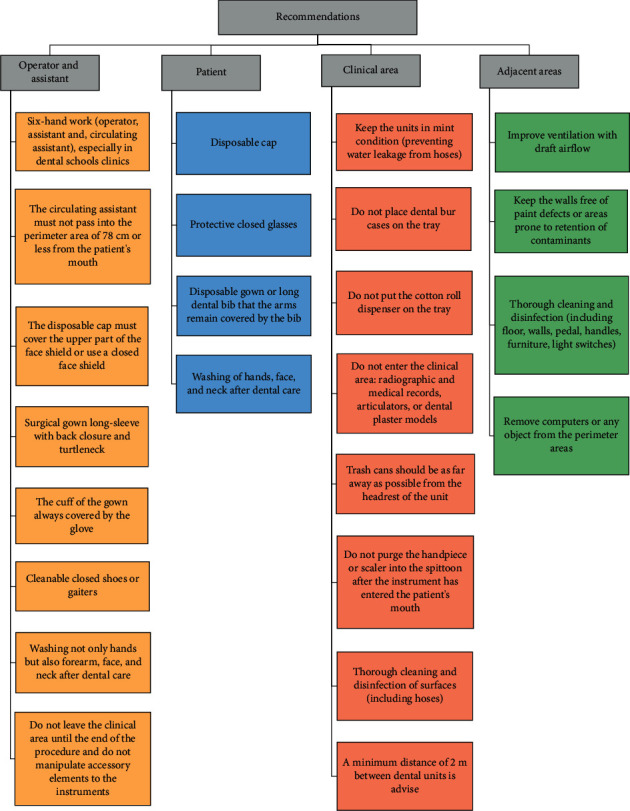
Recommendations based on outcomes. The recommendations above complement those widely reported in the literature and those disseminated by health regulators in different countries.

The recommendations suggested based on the findings of the current study are shown in Figure 12. These recommendations complement those that were widely disseminated in previous reports [11, 12, 25–28].

## 4. Discussion

The results of the present study provide evidence that aerosol particles generation is an imminent consequence of carrying out dental procedures and constitutes a potential mechanism for the spread of several infections such as that produced by SARS-CoV-2. The spread of aerosol particles during AGPs represents a significant risk of exposure, primarily for dental staff, if biosecurity and cleaning and disinfection measures are not responsibly adopted. The variables that were associated with a higher risk of exposure in the prediction model were as follows: a distance of less than 78 cm, low ventilation, the use of a high-speed handpiece or pneumatic scalers (in periodontics)—with irrigant flow rates calibrated to make them comparable with each other; the location of the patient, operator, and assistant; and, to a lesser degree, the intervention of the anterior region of the mouth. The operator, assistant, and patient resulted consistently stained by the emanated particles.

The majority of the settled particles generated during the AGPs presented stained areas ranging from 1 to 5 *µ*m. This size has been previously associated with increased severity, morbidity, and fatality in infected patients because droplet nuclei can penetrate the respiratory tract to establish infection in the lower airways [29]. In the case of SARS-CoV-2 infection, droplet nuclei seem even more risky insofar as the epithelial cells of the lung alveolar surface abound with the angiotensin-converting enzyme II (ACE2), which works as a receptor for the S protein present on the surface of this virus [30].

The association of these variables should be put into the clinical context, considering the reported transmission routes of SARS-CoV-2, either by direct contact or by airborne transmission [11, 12]. The proximity between the patient—possibly infected—and the dental staff poses a risk of contagion, which is dependent on the adherence to the biosafety standards and the use of the recommended PPE [9, 12, 19]. Aerosol particles of different sizes, mainly <5 µm (86%), were produced. Some of them may settle due to gravity, whereas some could remain suspended in the air and enter the respiratory tract [31], which favors the spread of SARS-CoV-2 insofar as its dissemination is not produced exclusively via airborne transmission or droplet mechanisms but by both methods simultaneously [19], with an added risk in dentistry due to the high transmissibility of the virus during the asymptomatic period [32].

The permanence of these aerosol particles suspended in the air depends on the environmental conditions [33]. The infectious range depends mainly on the time interval between its presence in the atmosphere until its settlement [23]. Factors such as relative humidity, ambient temperature, and airflow have been closely related to the particle size and the time it takes to settle on a surface [33]. During sample collection, conditions of 70% relative humidity and a temperature of 20°C could favor the settlement of the aerosol particles. Previous studies have shown that low relative humidity [16] and high ambient temperature [33] are related to a longer residence time of the droplet nuclei and droplets in the air [34]. The two environmental conditions mentioned increase the tendency of the drops to pass to the vapor phase, which tends to decrease their size by drying. This results in an increase in the mobility and circulation of the particles in the air [35], thereby increasing the risk of spreading the infection in the clinical area [36].

Poor ventilation demonstrated a high association with a greater stained area. In addition, previous reports estimated that better ventilation substantially reduces the suspension time of aerosol particles in the air [37]. The positive influence of ventilation will depend on several conditions: first, on the amount of outdoor air that is available within the indoor space, defined as the ventilation rate; second, the direction of airflow from clean areas toward contaminated areas; and, finally, the distribution of air, which must cover all spaces while entering and leaving the clinical area [38]. These characteristics will depend on the infrastructure and layout of the area [39]. In this study, the air-exchange rate with open windows allowed air renewal that complies international standards [40]. Although in this study, the experiments were carried out in six different clinical situations and twelve different dental unit locations, the extrapolation of the results should be done with caution, protecting the staff from exposure to hazardous conditions using engineering control measures, and without disregarding the particular layout of each setting [41].

The methodology used in this study evaluated the size of the area stained by the drops that settle on the paper, which does not necessarily correspond to the diameter of the aerosolized particles. This methodology has been used in numerous studies [42–44]. Other methodologies used include the use of particle image velocimetry where Li et al. [45] report that most of the particles generated by ultrasonic scaler have a size between 50 and 180 *µ*m. On the other hand, Plog et al. [46] reported 43 *µ*m particles for the ultrasonic scaler, using the blacklight (LED) shadowgraph method. In the present study, stained areas produced by particles generated by the ultrasonic scaler were obtained from 1 *µ*m to more than 10 *µ*m, some reaching up to 180 *µ*m. It is evident that the size of the generated particles depends on the working conditions, such as the irrigation flow rate, ventilation, and airflow, added to the measurement method.

The mass of the aerosol particles determines several settlement patterns resulting from different sizes and shapes of the aerosol particles deposited on the surfaces [47]. Sedimented particles may facilitate the transmission of infection by fomites [10]. Thick drops may be formed by splashes produced by the rebound of the pressurized water on some oral structure or by the accumulation of oversized droplets on the operator's gloves or the patient's face and neck, which means that a mixture of aerosol particles with particles that are not aerosol particles [19], which can contain saliva, blood, and microorganisms [10], might have caused some portion of the stained areas. Furthermore, thick droplets may be formed by the phenomenon of coalescence or aggregation [19, 47], defined as a binary process in which two drops of the liquid merge to form a single drop. The factors that directly influence drop-drop interactions include Brownian motion, viscosity, density, interfacial contact area, diffusivity, surface tension, and concentration gradients; therefore, this interaction depends on the nature of the liquids [48–50].

As reported by Guzman [51], the SARS-CoV-2 viral load required to initiate COVID-19 disease is expected to be below 1,000 particles. In theory, taking into account the size of a SARS-CoV-2 viral particle is in the range of 0.006–0.14 *µ*m [52], a 1 *µ*m drop could transport around eight viral particles. Hence, any aerosol particle or set of aerosol particles over 120 *µ*m in size may contain sufficient viral load for infection. Of the 1256 samples obtained in the current study, 664 presented stained areas ≥120 *µ*m, which makes transmission via generated aerosol particles biologically plausible during a dental procedure. However, other factors, such as the infectious capacity of the virions in the particles [29, 53], the inactivation potential of the virus, the saliva-water dilution ratio that varies between 1 : 20 and 1 : 100 [19], the chemical composition of the drops, and the viability on different surfaces [54, 55], should be taken into account when evaluating the infectious potential of the aerosol particles.

Different methods like luminescent tracer [43, 56], particle image velocimetry [45], blacklight (LED) shadowgraph [46], microbiological analysis [57], and spectrofluorometric analysis [43, 44] have been employed to study the spatial distribution of droplets and aerosol particles; our findings were consistent with these previous studies, despite having used a different method [58, 59]. Our methods were able not only to characterize the risk of exposure when performing the AGPs by using the settlement patterns of the particles generated during the procedures but also to recognize potential contamination sources within a dental care setting, and to delimit critical areas for the settlement of particles. Our findings are also useful for guiding the implementation of new clinical techniques in dental operatory and new teaching models in dental schools, as well as for evaluating the effectiveness of ventilation and extraction systems and PPE kits.

The present study has the following limitations. First, an *in vivo* model was not used to determine the amount of viable infectious viruses in the aerosol particles. Second, the model used in this study was sensitive and was able to detect only particles that have the capacity by size and weight to settle during the 30-minute period after the completion of the AGPs, which has been reported as the time through which most of the aerosol particles are likely to settle [3, 58, 60]. Therefore, another model will be necessary to determine the amount of viable infectious viruses remaining in aerosol particles, as well as the amount and size of aerosol particles that remain suspended in the environment for a longer period. Third, the method used in this study does not allow us to assure any correspondence between the aerodynamic diameter of each particle and the area stained by their settlement; therefore, the outcome variable represents the stained area on the filter paper and not the size of aerosolized particles. Last, the saliva pooled in the posterior region of the oral cavity of the mannequins does not accurately simulate normal saliva flow.

Nonetheless, a significant contribution was made to the characterization of the size and settlement patterns of the aerosol particles generated by widely used instruments in dental school clinics and health care settings and, consequently, to the determination of specific biosafety measures proven to be effective for protecting both the dental staff and the patient from the infection risk associated with the dynamic behavior of aerosol particles generated during dental AGPs. Even though the estimated infection rate among dental care workers during the first waves of the pandemic ranges between 1% and 10% [4], resuming activities at dental care settings is likely to cause this amount to rise in case biosafety measures are overlooked.

## 5. Conclusions

Under the limitations of this study, the highest percentage of the stained area by settled particles ranged from 1 to 5 *µ*m. The settlement patterns of the aerosolized particles varied depending on the area surrounding the phantom head and the instrument used. Depending on the stained area by settled particles, they could be classified into ultrafine-fine and thick drops, which showed point, splatter, and spot patterns. The variables associated with the stained area outcome were distance, ventilation, the instrument used, location in the perimeter area, and area of the mouth to be treated. Settled particles were able to reach a distance of 320 cm away from the mouth of the phantom head, with a cutoff point of 78 cm as a risk factor. Thus, our model is useful insofar as it included several variables to develop a prediction scale for estimating the risk of exposure to an airborne virus like SARS-CoV-2 associated with the dynamic of the settlement of the particles generated during AGPs.

## Figures and Tables

**Table 1:** Comparative analysis between the stained area for the pneumatic scaler and the ultrasonic scaler.

Type of scaler	*N*	Min^*∗*^	Max^*∗*^	Median^*∗*^	Rank^*∗*^	Mean^*∗*^	SD^*∗*^	*P* value (<0,05)
Pneumatic	100	0	10758	1972	10758	305.5	1294	0,0084
Ultrasonic	100	0	2507	8,3 × 10^−6^	2507	97.6	381.6	

^*∗*^Stained area data in *µ*m.^2^.

**Table 2:** Sizes of the stained area of settled particles.

Size	Average of settlement^*∗*^
1 and 5 *µ*m	86%
5 and 10 *µ*m	4.2%
>10 *µ*m	9.8%

^*∗*^Mean particle settlement percentage throughout the eight procedures performed per clinic, according to the size of the stained area of the settled particles.

**Table 3:** Association of independent variables.

Independent variable	Condition of care	*P* value
Division	With modular division between units	0.45
	Without modular division between units	

Dental unit	1 (adequate ventilation)	0.08
	2 (inadequate ventilation)	

Tooth	Anterior	0.06
	Posterior	

Ventilation	Open	0.020^a^
	Close	

Distance	>78 cm	0.001^a^
	<78 cm	

Instrument	High-speed handpiece	0.001^a^
	Pneumatic scaler	
	Ultrasonic scaler	
	Triple syringe	

Filter paper location	Operator and patient	0.001^a^
	Assistant	
	Dental equipment	
	Accessory surfaces	
	Adjacent surfaces	

^a^Statistical association of independent variables (*P* < 0.05).

**Table 4:** Cutoff points of greater discrimination for the associated variables.

Variable	p25	p50	p75	Min.	Max.	Mean	SD	*N*
Distance	40	78	136	15	320	98	66	1256
Stained area^*∗*^	0	3.4	69	0	5260900	12274	208.3	1256

^*∗*^Value in *µ*m^2^. The total area of the filter paper was 6′358,500 *µ*m^2^. Max. stained area: 82.7%.

**Table 5:** Prediction model of the risk of infection due to exposure to aerosol particles generated during AGPs.

Variables	OR		IC 95%	*P* value
Tooth	1.1		(0.8–1.5)	0.4
Ventilation	1.5		(1.1–2.0)	0.001^∗^
Distance	2.7		(1.8–3.8)	0.001^∗^
Instrument	1	1	Reference	0.001^∗^
Filter paper location	1	1	Reference	0.001^∗^
	2	1.3	(1.2–3.5)	0.001^∗^

^*∗*^ Variables with statistically significant difference were considered as a risk factor. Instrument 1 (high-speed handpiece) and filter paper location 1 (operator and patient) were taken as the basal level (reference) with an OR = 1.

**Table 6:** Score of the variables according to the OR.

Variable		OR	Score
Tooth	Anterior	1.1	1
Ventilation	Closed	1.5	2
Distance	<78 cm	2.7	3
Instrument	Handpiece	1	1
Location	Operator and patient	1	1
	Assistant	1.3	2
Total			10

**Table 7:** Scale validation in terms of discrimination and goodness of fit.

Score	Prob	Obs 1	Exp 1	Obs 0	Exp 0	Total
1	0.0588	3	4.7	133	131.3	136
2	0.0943	18	10.3	114	121.7	132
3	0.1370	13	13.2	101	100.8	114
4	0.2665	26	24.0	96	98.0	122
5	0.4575	34	45.0	91	80.0	125
6	0.6291	71	71.6	57	56.4	128
7	0.7315	86	85.9	39	39.1	125
8	0.7953	88	93.8	35	29.2	123
9	0.8496	107	105.7	21	22.3	128
10	0.9261	116	107.6	6	14.4	122

**Table 8:** Sensitivity and specificity values of the scale.

Classified + if predicted Pr (D) ≥ 5 True *D* defined as area = 0	
Sensitivity	Pr (+|D) 80.96%
Specificity	Pr (-|∼D) 78.64%
Positive predictive value	Pr (D| +) 75.46%
Negative predictive value	Pr (∼D| -) 83.59%

**Table 9:** Prediction scale of the risk of infection due to exposure to aerosol particles generated during AGPs.

Variable		Score
Tooth	Anterior	1
	Posterior	0

Ventilation	Open	0
	Close	2

Distance	<78 cm	3
	>78 cm	0

Instrument	Handpiece	1
	Other	0

Location	Operator and patient	1
	Assistant	2

Total		10

## Data Availability

The datasets used during the current study are deposited in Mendeley Data, V1, doi: 10.17632/wvm3jmdy98.1 (https://data.mendeley.com/datasets/wvm3jmdy98/1).
